# Supplementary material for: Bacterial Surface Disturbances Affecting Cell Function during Exposure to Three-Compound Nanocomposites Based on Graphene Materials
Source: Nanomaterials (Basel). 2022 Sep 2;12(17):3058. doi: 10.3390/nano12173058 (PMC9459733; doi:10.3390/nano12173058)
Supplement: Supplementary file 1 [file nanomaterials-12-03058-s001.zip › nanomaterials-1859867-supplementary.pdf]

nanomaterials

MDPI

Supplementary Materials

Bacterial Surface Disturbances Affecting Cell Function during Exposure to Three-Compound Nanocomposites Based on Graphene Materials

Agata Lange [1,*], Ewa Sawosz [1], Karolina Daniluk [1], Mateusz Wierzbicki [1], Artur Małolepszy [2], Marcin Gołębiewski [3] and Sławomir Jaworski [1,*]

[1] Department of Nanobiotechnology, Institute of Biology, Warsaw University of Life Sciences, 02-786 Warsaw, Poland
[2] Faculty of Chemical and Process Engineering, Warsaw University of Technology, 00-654 Warsaw, Poland
[2] Department of Animal Breeding, Institute of Animal Sciences, Warsaw University of Life Sciences, 02-786 Warsaw, Poland
* Correspondence: agata_lange@sggw.edu.pl (A.L.); slawomir_jaworski@sggw.edu.pl (S.J.);
Tel.: +48-225936675 (S.J.)

Citation: Lange, A.; Sawosz, E.; Daniluk, K.; Wierzbicki, M.; Małolepszy, A.; Gołębiewski, M.; Jaworski, S. Bacterial Surface Disturbances Affecting Cell Function during Exposure to Three-Compound Nanocomposites Based on Graphene Materials. *Nanomaterials* **2022**, *12*, 3058. https://doi.org/10.3390/nano12173058

Academic Editors: Maria Letizia Manca and Heyou Han

Received: 26 July 2022
Accepted: 30 August 2022
Published: 2 September 2022

Publisher's Note: MDPI stays neutral with regard to jurisdictional claims in published maps and institutional affiliations.

Copyright: © 2022 by the authors. Licensee MDPI, Basel, Switzerland. This article is an open access article distributed under the terms and conditions of the Creative Commons Attribution (CC BY) license (https://creativecommons.org/licenses/by/4.0/).

Analysis of zeta potential dependence on pH

Zeta potential was measured by electrophoretic light scattering (ELS) using the Zeta Sizer Nano-ZS90 analyzer (Malvern Instruments, Malvern, UK) at room temperature for all nanomaterials, preceded by sonication at 500 W and 20 kHz for 2 min, and in case of nanocomposites, they were left for 15 min for self-combination. Each nanosuspension was measured in 3 different pH values (4, 7 and 10).

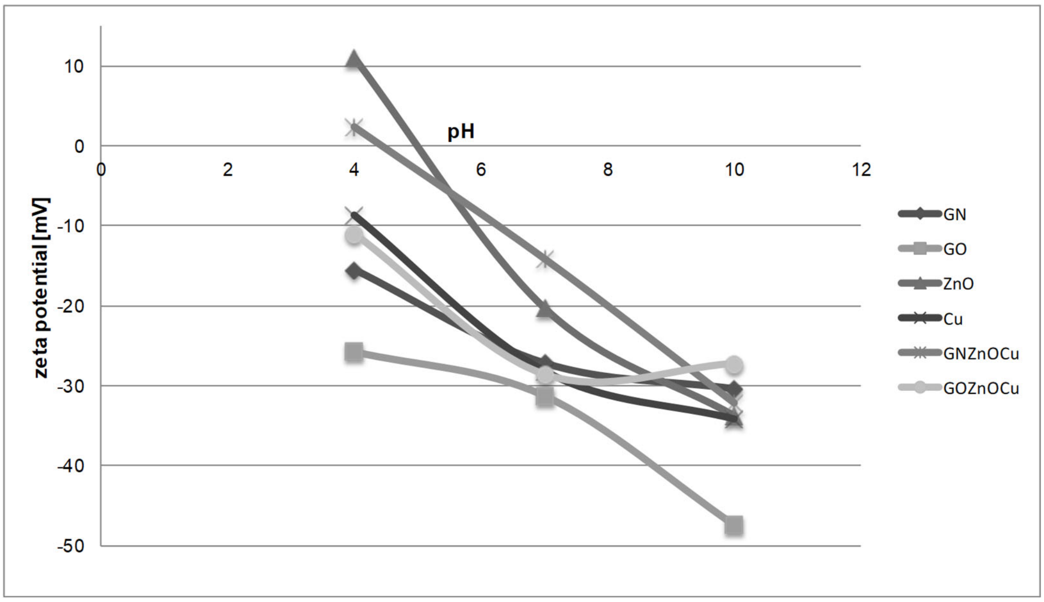

Figure S1. Zeta potential of nanomaterials tested in different pH values. Each measurement was performed in triplicate.

pH analysis of nanoparticles in culture medium

The pH measurements of nanomaterials in Mueller–Hinton broth medium (Biomaxima, Lublin, Poland) were measured with the use of the pH meter SI Analytics HandyLab 100 (Xylem Water Solutions, Washington, WA, USA). Each measurement was conducted in two time points: immediately after added nanoparticles into medium (0h) and after 24 hours of incubation (24h). The analysis was performed in triplicate at the room temperature.

Table S1. pH values of nanomaterials tested in culture medium in two time points (0h and 24h). Results are presented as a mean values ± standard deviation. C is control medium without nanoparticles.

Nanomaterials	0h	24h
C	7.29 ± 0.11	7.29 ± 0.01
GN	7.28 ± 0.02	7.28 ± 0.01
GO	7.28 ± 0.03	7.28 ± 0.01
ZnO	7.33 ± 0.02	7.36 ± 0.01
Cu	7.29 ± 0.02	7.30 ± 0.01
GNZnOCu	7.32 ± 0.02	7.31 ± 0.01
GOZnOCu	7.31 ± 0.02	7.31 ± 0.01
